# Patient experiences of switching from Efavirenz- to Dolutegravir-based antiretroviral therapy: a qualitative study in Uganda

**DOI:** 10.1186/s12879-021-06851-9

**Published:** 2021-11-13

**Authors:** Adelline Twimukye, Miriam Laker, Eva Agnes Laker Odongpiny, Florence Ajok, Henry Onen, Ivan Kalule, Phoebe Kajubi, Kay Seden, Noela Owarwo, Agnes Kiragga, Mari Armstrong-Hough, Anne Katahoire, Andrew Mujugira, Mohammed Lamorde, Barbara Castelnuovo

**Affiliations:** 1 Infectious Diseases Institute, College of Health Sciences, Makerere University, P. O Box 22418, Kampala, Uganda; 2 Uganda Red Cross, Kampala, Uganda; 3 University of Liverpool, Liverpool, UK; 4 School of Global Public Health, New York University, New York, NY USA; 5 Uganda Tuberculosis Implementation Research Consortium, Makerere University, Kampala, Uganda; 6 Center for Interdisciplinary Research on AIDS, Yale University, New Haven, CT USA; 7 School of Public Health, College of Health Sciences, Makerere University, Kampala, Uganda

**Keywords:** HIV, Dolutegravir, Drug switching, Qualitative research, Uganda

## Abstract

**Background:**

In 2019, the World Health Organisation (WHO) recommended Dolutegravir (DTG) as the preferred first-line antiretroviral treatment (ART) for all persons with HIV. ART regimen switches may affect HIV treatment adherence. We sought to describe patient experiences switching from EFV to DTG-based ART in Kampala, Uganda.

**Methods:**

Between July and September 2019, we purposively sampled adults living with HIV who had switched to DTG at the Infectious Diseases Institute HIV clinic. We conducted in-depth interviews with adults who switched to DTG, to explore their preparation to switch and experiences on DTG. Interviews were audio-recorded, transcribed and analysed thematically using Atlas ti version 8 software.

**Results:**

We interviewed 25 adults: 18 (72%) were women, and the median age was 35 years (interquartile range [IQR] 30–40). Median length on ART before switching to DTG was 67 months (IQR 51–125). Duration on DTG after switching was 16 months (IQR 10–18). Participants reported accepting provider recommendations to switch to DTG mainly because they anticipated that swallowing a smaller pill once a day would be more convenient. While most participants initially felt uncertain about drug switching, their providers offer of frequent appointments and a toll-free number to call in the event of side effects allayed their anxiety. At the same time, participants said they felt rushed to switch to the new ART regimen considering that they had been on their previous regimen(s) for several years and the switch to DTG happened during a routine visit when they had expected their regular prescription. Some participants felt unprepared for new adverse events associated with DTG and for the abrupt change in treatment schedule. Most participants said they needed additional support from their health providers before and after switching to DTG.

**Conclusion and recommendations:**

Adults living with HIV stable on an EFV-based regimen but were switched to DTG in a program-wide policy change found the duration between counselling and drug switching inadequate. DTG was nonetheless largely preferred because of the small pill size, once daily dosing, and absence of EFV-like side effects. Community-engaged research is needed to devise acceptable ways to prepare participants for switching ART at scale.

**Supplementary Information:**

The online version contains supplementary material available at 10.1186/s12879-021-06851-9.

## Background

Dolutegravir (DTG), an integrase strand transfer inhibitor, has better efficacy, tolerability and durability than non-nucleoside reverse transcriptase inhibitor (NNRTI)-based first-line regimens [1]. DTG is recommended by the World Health Organization (WHO) as the preferred first- and second-line antiretroviral therapy (ART) [2–5]. DTG induces rapid viral suppression following ART initiation, and has a higher genetic barrier to drug resistance and lower potential for drug-drug interactions than efavirenz (EFV) or nevirapine (NVP) [5]. In 2017, the WHO recommended transitioning to DTG-based first line regimens in settings where pre-treatment NNRTI drug resistance exceeds the recommended 10% threshold, such as in East and Southern Africa [6]. There is complexity in dealing with HIV related drug switches, including management of drug induced toxicities, risk communication and response to side effects. Since DTG is a relatively new drug, information about user experiences and management of side effects as well as the communication of DTG risks is critical to optimising wider rollout.

By mid-2019, the national ART programmes of most low- and middle-income countries (LMIC) had adopted or planned to adopt a DTG-based regimen for first-line treatment [6]. Programmatic evaluations of tenofovir (TDF) switches have been conducted in three African countries, but were quantitative in nature [7]. While quantitative studies may be useful in addressing some switch gaps, they are survey based and provide limited data on participant experiences. There has been little evaluation of patient perspectives during drug switching in public health programs among HIV patients in Africa. Adverse events are commonly associated with drug switching [8], but little is known about other patient experiences during the switch to DTG. Numerous challenges to drug switching are driven in part by the bottlenecks of the weak health systems of LMICs and short-time lines that have been set for transitioning patients to DTG-based ART [9]. When providers adopt guidelines, participants may experience new challenges to adherence after switching from EFV or NVP-based ART to DTG-based ART [6]. In the Swiss HIV Cohort Study, up to 6% of patients discontinued DTG after drug switching because of neuropsychiatric side-effects [10].

In 2018, WHO released interim guidelines recommending a cautious approach to DTG use in women due to reported potential risks in pregnancy, including neural tube defects (NTDs) when used in the preconception period [11]. DTG usage was restricted to women who were using effective contraception or already in the second or third trimester of pregnancy. However, in 2019, WHO updated its guidance for women to use DTG without use of contraception or pregnancy testing [6]. Educating women about potential risks and benefits of DTG might cause women to make informed decision about accepting or rejecting DTG. All restrictions for use of Dolutegravir-based regimen as preferred first- and second-line ART for all pregnant and breastfeeding women were removed from Uganda treatment guidelines [12]. The 2020 HIV guidelines recommend procedures for substituting ARVs in adults, adolescents and children already on first line ART and recommend options for subsequent second and third-line regimens. These guidelines emphasize the importance of pharmacovigilance (PV) and describe the procedures for identifying, investigating, reporting and management of adverse effects of ART, anti-TB and other medications. Importantly, the Uganda Ministry of Health (MoH) trained health care providers on DTG switching.

In Uganda, participants were switched in phased manner in 2018 to the newly recommended first-line regimen—tenofovir, lamivudine and dolutegravir (TLD)—for adults weighing ≥ 35 kg [13]. It was estimated that 250,000 eligible men and women would have accessed TLD by December 2019. In Uganda, approximately 1.3 million people with HIV are on ART [14]. Prior to the national rollout of DTG, the Uganda MoH and the Clinton Health Access Initiative (CHAI) launched a DTG pilot study in July 2017 for persons newly initiating treatment or switched from an NNRTI regimen due to intolerance [15]. At the time of the study, little was known about the experiences of people with HIV when their provider switched them to DTG. Few published studies have examined patient experiences of being switched to DTG [6]. Qualitative studies can help program designers, implementers and policy makers better understand patient experiences during the implementation of new treatment policies [11]. Describing patient switch experiences is critical to inform programs and policy decisions that may promote uptake and adherence to DTG-based treatment [16]. Therefore, we aimed to describe participants’ experiences switching from EFV-based and NVP-based ART to DTG-based ART at a large urban HIV clinic in Kampala, Uganda.

## Methods

### Setting

The Infectious Diseases Institute (IDI) HIV clinic is a large urban care centre with more than 8000 clients on ART. It consists of a general clinic (with free clinical services) which operates from 8:00am to 5:00 pm and a co-pay clinic, an after-hours clinic which operates after 5:00 pm, that offers patients options for voluntary financing of some health care services in return for more convenient services [17]. In July 2017, the Uganda MoH selected the IDI as an early adopter site to pilot DTG-based ART. The purpose of the pilot study was to generate preliminary data to inform national scale-up. Drug switching in the pilot took place in two phases: from July 2017, participants on first line NNRTI-based ART (commonly tenofovir, lamivudine and efavirenz; TLE) who had achieved viral suppression but were experiencing toxicities were switched. From March 2018, other patients who were all virally suppressed participants on first-line NNRTIs not experiencing side effects were also switched.

HIV counsellors used a standard operating procedure (SOP) provided by the Uganda MoH to explain the DTG switch to eligible participants. The SOP required providers to explain the switch to DTG-based ART (TLD), how to take DTG, possible benefits of drug switching, side effects, and other considerations. Participants were educated about the change in routine for taking DTG in the morning hours to avoid insomnia. Health talks and discussions about DTG switch were conducted at the clinic waiting area with groups of participants who had come for their routine visits. Health talks were given early in morning at the general clinic and not at the evening co-pay clinic. Participants were not told in advance of their routine visit that their ART regimens were to be changed. Counsellors assessed patient readiness to switch by asking the individual whether they were ready to switch or not. For most participants, drug switching occurred on the same day they were counselled.

### Study design

Between July and September 2019, we conducted a qualitative study using a phenomenological approach at the IDI HIV clinic in Kampala, Uganda.

### Sampling

In July 2019, using a maximum variation strategy, we purposively sampled 25 adults aged ≥ 18 years with HIV who switched to DTG from a first-line regimen. Eligible participants had to have been on ART at the IDI HIV clinic for more than one year before they were switched to DTG. Participant selection took into consideration age, gender, marital status and socio-economic status.

### Data collection

Potential participants were invited via phone or personal contact to participate in a face-to-face in-depth interview conducted in a private location at the IDI HIV clinic. The topic guide included open-ended questions and flexible probes to investigate the personal switch experiences of each respondent. We asked open ended questions about: (i) perceived risks and benefits of switching to DTG), (ii) switch preparation process, (iii) experiences at the time of switch, (iv) treatment experiences on DTG, and (v) suggestions to improve the switching experience. We conducted interviews in one of two languages (English and Luganda [local language]) depending on the participant’s preference. The sample size was determined by saturation [18] meaning new participants were recruited until no new themes emerged. Interviews were audio-recorded with participant consent and transcribed verbatim for data analysis. Transcripts were checked for completeness and accuracy. The study also collected baseline information for the study participants.

### Data management and analysis

We used an inductive approach to apply descriptive, thematic codes [19] using ATLAS.ti, version 8 (Berlin, Germany). Transcript recordings and notes were reviewed for content related to the research question and a coding scheme developed. The initial codebook was developed after careful line-by-line analysis of the text in each transcript through open coding. We assigned codes to relevant segments of the text, and similar or related codes aggregated to form themes [9] such as positive and negative experiences of drug switching, experiences with DTG-related side effects and to develop coherent narratives. We report key phrases and verbatim quotes on the switch experience, organized by theme. The consolidated criteria for reporting qualitative research (COREQ) checklist was used to report study findings [20].

### Ethical approval

This study was approved by the Joint Clinical Research Center Research Ethics Committee (JC3118), and by the Uganda National Council for Science and Technology (SS 314ES). A bilingual Luganda and English speaker carried out forward- and back-translation of the consent form. The research assistants (RAs) read the consent form aloud, and all participants provided written informed consent.

## Results

### Participant characteristics

We used purposive sampling to approach 30 participants to take part in IDIs and interviewed 25. Five participants were not interviewed because of lack of time due to their busy work schedules. Participants included ten women of reproductive age or older and ten men. We also included five women of reproductive age who had ever used DTG but stopped due to pregnancy restrictions. However, interviews among women of reproductive age were conducted after the release of the new data regarding possible DTG safety risks in pregnancy. Of the 25 participants, four were from the co-pay clinic, of higher socio economic status and not directly comparable to those in the general clinic. Most participants (68%) were women (Tables 1, 2). Median age was 35 years (interquartile range [IQR] 30–40). Median length on ART before drug switching was 67 months (IQR 51–125). Duration on DTG after switching was 16 months (IQR 10–18). The majority (56%) described themselves as married or cohabiting and as having completed ≥ 8 years of education (68%). Nearly one-in-three (28%) had WHO stage 3 HIV disease and five (20.8%) self-reported experiencing poor adherence after drug switching to DTG (Table 1).

**Table 1:** Baseline characteristics for 25 participants switched to DTG

Characteristic	N (%) or Median (IQR)
Age, years	35 (30—40)
Duration on ART prior to DTG switch, months	67 (51—125)
Duration on DTG after switching, months	16 (10—18)
Gender	
Female	17 (68)
Male	8 (32)
History of toxicity	
Stable on DTG	1 (4)
No history of toxicity	18 (72)
Experienced toxicities	6 (24)
Education level, years	
Never been to school (illiterate)	3 (12)
Primary (≤ 7)	5 (20)
Secondary (8–13)	9 (36)
Post-secondary (> 13)	8 (32)
Marital status	
Married/living with partner	14 (56)
Separated/divorced	1 (4)
Single	7 (28)
Widowed	3 (12)
WHO stage	
Stage 1	5 (20)
Stage 2	7 (28)
Stage 3	7 (28)
Stage 4	6 (24)
Adherence history	
Good adherence (took drugs daily and on time)	20 (80)
Poor adherence (failed to take drugs daily and or on time)	5 (20)

*IQR* interquartile range

**Table 2:** Patient categories by gender

Participant category	Participants interviewed	
	Male	Female
Participants stable on DTG (SOD)	5	5
Participants who experienced side effects (ESE)	3	2
Women of reproductive age on DTG (RSD)	0	5
Women of reproductive age off DTG (RSOD)	0	5
Total	8	17

Some patient categories overlap due to multiple experiences. Some participants were peer experts who had participated in switch decision meetings as well as participants of high economic status who attended the IDI-Co-pay evening clinic

**Fig. 1:**
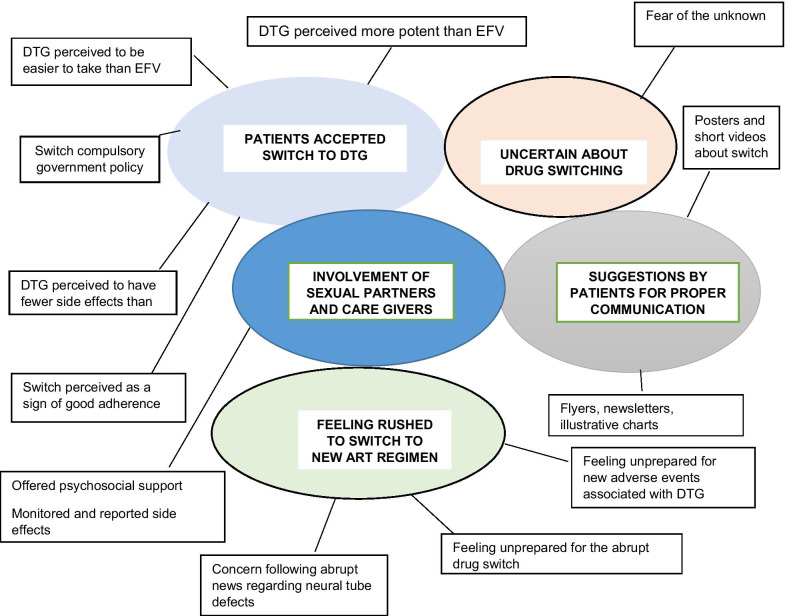
Thematic map showing five main themes from participants switched to DTG

We identified five major themes in the data: (1) patients accepting the switch to DTG, (2) uncertainty about drug switching, (3) feeling rushed to switch to a new ART regimen, (4) involvement of sexual partners and care givers in patients rushed decision to switch, and (5) suggestions by patients for proper communication and effective orientation to new treatment option which are represented in Fig. 1**.**

## Reasons for accepting the switch to DTG

### DTG dosing perceived to be simpler than EFV

Participants said they accepted their provider’s recommendation to switch to DTG in part because they anticipated that swallowing a smaller pill just once a day would be more convenient than their past regimen especially for those who had been taking nevirapine regimens that were dosed twice daily. Common terminologies that ran through participants narratives included: “…it is easy to swallow”, “… you can swallow it without eating”, “…this one (DTG) reduces on the side effects of the previous one”, “…smaller pill as compared to efavirenz”, “… It is one tablet so it is not difficult”, ‘’…I swallow it once’’. They found DTG convenient and easier to take than EFV because it is smaller and taken less frequently:

I was like, now why are they changing me to this drug [laughter], but I had to accept it. Right now, I feel the benefit is much more, I feel switch is worth it; … Its smart taking! I just take once in the morning… at nine in the morning and that is it. [IDI_male patient_co-pay_stable on DTG (SOD) _16]

### DTG perceived to be more potent than EFV

Participants perceived DTG as a new drug innovation that was more efficacious compared to their previous EFV-based regimen because was the newest ART introduced that rapidly suppressed the virus as commonly expressed: “… It is stronger,” “…it is the best at suppressing the virus”; “…it is an improvement:”

Changing it (EFV) and transitioning to another one like this one (DTG); I see that if one adheres well to his drugs… the virus is suppressed quickly. [IDI_female patient_ general clinic_SOD_17].

### DTG perceived to have fewer side effects than EFV

Majority of participants were encouraged by the preparatory health talk they received about DTG, which emphasized resolution of EFV-related side effects such as dizziness and hallucinations:

Hmmm, there are many changes, Musawo [doctor]; like I no longer feel the dizziness and hallucinations I used to... I have no concern at all because even now, I can swallow it in the morning, and I reach 12 noon or 1pm working, without having eaten anything. But before, I could not do it; I had to eat food before I swallow the drug. If I do not eat, I cannot even see where I am going. [IDI_male patient_general clinic_SOD_10]

### Switch perceived as a sign of good adherence

Most participants believed that their health providers flagged them as eligible for the switch because they had achieved viral suppression. For example, participants explained” *…*they decided to change me because my viral load was suppressed,” that the “… patient should have been doing well on the previous drug”, and that “… There was a tag on my file recommending me on the new drug’’. Participants felt that simplifying the drug regimen was a reward for those taking their first line drugs as prescribed and on time and a sign that they were good participants:

The reason they gave me for switch was that I was adhering well to the ART. The doctors flagged my file because I qualified to start DTG. If your file has a good record of good adherence, low viral load; in other words, you are a good patient. If they follow up your adherence record and it is good, they put you on DTG. [IDI_female patient_ general clinic_SOD_3].

### Feeling that drug switching was a compulsory government policy

The majority of participants felt they had to accept switching because it was a compulsory government policy. It was mandatory to switch every client who was on first-line ART in accordance with new MoH guidelines:

I heard it was a government policy. So, it was a must! No negotiations! I had no decision to make. As I said at the beginning this was a government policy and when change comes, I just go with the flow. [IDI_male patient_co-pay clinic_ SOD_15]

## Uncertainty about drug switching

### Fear of the unknown

Most participants initially hesitated to switch to DTG. They worried because they felt uncertain about its related side effects:

… I was a bit scared thinking of why they would change my drugs, wondering what could have happened… however, afterwards I was like, “Ah, these are the people that treat us every day so they cannot put us on a drug that can cause harm. [IDI_female patient_ general clinic_SOD_6]

All participants said their providers offered frequent appointments and the opportunity to consult them using a toll-free number if they experienced side effects in order to allay uncertainty about drug switching:

He even gave me a few months so that I can come back quickly in case I get any problem. I said, ‘Why have you given me few months?... we shall give it to you for a short time, to see how the drugs will treat you, and even if the time we gave you has not yet reached, you can come and tell us if you have experienced any challenges on this drug.’ Eh, then I asked him, ‘Do you expect me to experience some challenges?’ and he said "No, but in case there is any… [IDI_female patient_co-pay clinic experienced side effects (ESE) _7]

## Feeling rushed to switch to a new art regimen

The majority of participants said they felt rushed to start the new ART regimen that was prescribed during a routine visit when they had expected to pick up their previously prescribed medicines. They described the DTG switch process as “abrupt and surprising”:

It was a surprise to me because when I came here, I thought I was going to get the usual drug (EFV), only [for] the doctor to tell me, ’You bring out the drugs you have; then I picked it out from my handbag, then he said, ‘these ones (EFV) are remaining here’, am giving you a new drug (DTG)…. [IDI_female patient _co-pay clinic_SOD_25]

### Feeling unprepared for new adverse events associated with DTG

Few participants said there was no discussion of potential side effects when switched. The majority of participants said the doctors explained lack of sleep (insomnia) as the main side effect of DTG. Some participants felt surprised about other new adverse events associated with DTG, including insomnia, itching, blurred vision, vomiting, headache, and weight gain which made one participant to stop taking DTG (Table 3):

…. The doctor told me ‘it is good medicine’, it will quickly clear the virus and I said it is okay… you are the medic here.’ He made the decision and put me on the new drug (DTG). That is the only thing he told me… he did not explain the side effects… I felt like running mad… The doctor said, ‘ahh! Get her off… [IDI_female patient_ general clinic_ESE _23]

**Table 3:** Side effects experienced or reported by participants after DTG switch from previous regimen

Perceived DTG side effects	N = 25	Illustrative quote
Insomnia (trouble sleeping)	5 (20%)	I had no strong side effects except maybe insomnia for that entire first week; whether daytime or night time, I had no sleep… That is the only side effect I got… [IDI_female_ESE_2]
Weight gain	4 (16%)	Personally, I support it because it has helped me; my appearance has changed. Counsellor, I was small before but now! [Laughter]… it has worked for me I have gained weight… [IDI_female patient_ general clinic_SOD_3]
Dizziness	3 (12%)	… I do not know whether it is the drug or not; but physically I am not that very fine… I still have that dizziness, yeah. Like in the morning after taking that drug, after, like within that space of one hour, you feel there is something, as if you have taken an overdose… [IDI_female patient_ general clinic_SOD_8]
Nausea	2 (8%)	When I had started it… I used to get some nausea but I took it for a week and it stopped. [IDI_ female patient-general clinic_Reproductive age stopped DTG_(RSD)_21]
Loss of appetite	2 (8%)	… When I started on it I suffered side effects…, it first made me lose my appetite, I did not want to take water, but after a while, it stopped, and I continued taking it well. [IDI_female patient_ general clinic_SOD_3]
Diabetes	2 (8%)	…when some people swallow DTG, it causes diabetes. I heard one say that. [IDI_female patient_ general clinic_SOD_3]
Passing pink urine	2 (8%)	But my urine comes with that pink color; I don’t know whether it should be like that or it’s abnormal, I don’t know. [IDI- female_ patient_ SOD-_1]
Body weakness	2 (8%)	…if you take it when you don’t have substantial amount of water, there, it treats you badly, it makes one weak, you need to take it when you have taken substantial amount of water, [IDI_ female patient_RSOD_18]
Blurred vision	2 (8%)	Whenever I swallow it I get blurred eye sight, or when I swallow it the body becomes weak and I don’t sleep; things like that. [IDI_female general clinic_ESE_7]
Headache	1 (4%)	Got dizziness and headache when I had just started on it. But when I got used to it, I do not have any problem. [IDI_female patient_ general clinic_SOD_17]

Most participants said health talks about switching were done hurriedly because health workers were busy, which caused some participants to have knowledge gaps about DTG. The majority lacked sufficient information about DTG as commonly expressed,”… nobody told me about its side effects. I think people are so busy”, “…do not know switch from what drugs to what’’; “…I failed to get the name of the new drug”. All participants desired to know the reasons for drug switching, specifics of the new drug especially its related side effects, benefits compared to the previous regimen, timing, DTG use and pregnancy. All participants suggested information sharing mechanisms about switch such as patient leaflets, audio visuals and sensitisation seminars:

I wanted to know why they had changed it to this new drug. Was it any better compared to the other three...and the benefits of this new drug. Hmmm, was it leading us to the discovery? Because we are reducing, going on a new drug, maybe we shall start taking a half, then a quarter [laughter]. Yeah. I would have loved to hear that. [IDI_female patient_SOD_1]

Some participants especially those that attended the private co-pay clinic did not want to wait for long during health talks at the crowded HIV general clinic. Majority of participants suggested that such a switch required adequate education and preparation for a smooth switch:

I think you first prepare people other than coming from nowhere you and say I am changing… I think you prepare people, teach them about some developments and then maybe give them some examples of people who have taken it …I think they would appreciate that; and say… they are changing for the better; not for the matter of just changing it. [IDI_female_patient_SOD_1]

Few participants highlighted a concern about missing detailed health talks due to late coming and suggested health educators should educate at different times so that late comers may benefit:

Just that sometimes they start educating people early; participants who come early are the ones that get information. However, for us that come a bit late, sometimes the health workers have already taught… Most times if you do not come early, you do not even know what is going on. [IDI_female_patient_ Reproductive potential switched off DTG (RSOD) _24]

Participants who attended the evening clinic missed group health education about switching to DTG that only occurred only to participants who attended the day clinic:

With day clinic I saw the switch process was perfect because… they address them as a group, they tell them when they are together, the counsellor comes and talks to them, “we are changing this because of this and this”, which is not like in private clinic. [IDI_female_patient_SOD_8]

Most said they needed additional support from their health providers before and after switch such as additional extensive counselling:

... Keep counselling them because most people do things out of ignorance. Because if you give someone drugs and he reaches home then decides to ask himself as to why the doctors had to change his medication it doesn’t make sense... [IDI_male patient_SOD_14]

Participants sought social support from family members to help them cope with the switch.

I told my mother about the changed drugs and she told me it is not bad, it’s the same drug she is on; they had changed for her as well. She is on one pill; she didn’t get any problem with it that’s what she told me... [IDI_SOD_male patient _14]

### Concern following abrupt news regarding neural tube defects of DTG in pregnancy

There was widespread concern among participants about potential congenital abnormalities with DTG following foetal exposure to DTG during the first trimester of pregnancy. Most participants disapproved of switching to DTG during pregnancy and said health providers should dispel rumours about neural tube defects for them to accept DTG in pregnancy. Some women of reproductive age who were not using contraception said they chose to stop taking DTG:

I hear about the neural tube thing and especially about us girls …women who have not yet given birth, it is somewhat risky, and it is what I heard. So, I decided to change back to tenofovir, lamivudine, efavirenz (TLE). [IDI_female patient_ general clinic_RSOD_12]

### Feeling unprepared for the abrupt change in schedule required for DTG

Some also felt unprepared for the abrupt change in dosing schedule required for DTG. Participants were used to taking medicines at night, but with DTG, they switched to morning dosing to avoid insomnia and many struggled to adhere to the new schedule. A limited number of participants missed taking their medication on time by one to two hours because of the switch:

Again the dislike of taking it at daytime… I usually take this medicine at 10am; so in case they call me at 9am to go and start the training and it goes beyond 10am may be up to 11am; then it means… I missed for two hours. So, what I do at times when I know am going to train and my time will elapse, I take again at around 8am when am entering… at times you find when am still there without swallowing the medicine. [IDI_female patient_co-pay clinic_SOD_25]

In addition, timing fluctuated because of job schedules that made most participants cope by taking DTG at night instead of the recommended morning dosing as expressed by one participant:

At times, I would go for the morning run as my job requirement in a security company. The morning run, eeeh! You have to show up. Yeah! It is around five thirty, so six would find when you, when you are on the run, eeeh! So you cannot get off the parade come and take medicine…. So there, the job somehow compromised my adherence… [IDI_male patient_co-pay_SOD_16]

One patient said she missed doses for several days (3–4 days*)* due to failure to adapt to the new schedule:

I used to swallow the previous drug, EFV, at 10pm and this drug was to be swallowed in the morning… I took long without actually swallowing the drug in the morning. And honestly, I took about 3 to 4 days without swallowing those drugs because that is not the time I was used; I failed with that time… every time I would try to swallow it, time would just go, time would fly by. Honestly, the most I tried was twice but for other days, it failed… [IDI_female patient_ general clinic_SOD_17]

Five participants with detectable viral load were disappointed to see their viral load increase and attributed it to the rushed DTG switch. They were virologically suppressed before drug switching and felt the switch had interfered with this and suggested viral load monitoring prior to drug switching as illustrated in Table 4:

…I feel this medicine they gave me maybe it is not working in my body. What they would have done; I think before giving me that drug, they would have taken my viral load and see how it is, so that when am taking this one they could know whether it is the change of the drug, or it is my body. The body could be refusing this one… because the virus was undetectable prior to switch… Now today it has gone up after changing. So, I feel even this change of the medication affected me. [IDI_female patient_co-pay clinic_SOD_25]

**Table 4:** Interventions suggested by patients for a convenient drug switch

Intervention	Number that suggested intervention	Illustrative quote
Ensuring good doctor/clinician patient relationship	01	So, the drug substitution in IDI is sort of a teamwork, it is a decision, mutual understanding… It is not like, ‘ah, change!’ no, people will not accept forced switch… [IDI_male patient_ peer leader_ ESE_9]
Adequate preparation for switching to a new drug	15	… Okay, If I have three months, they can call me in the middle then they tell me your next appointment is supposed to be 30th, but we have called you today to tell you about what we may do on the 30th, then they tell me about the new drug… [IDI_male patient_ general clinic_ESE_11]
Home visits during a switch	01	So, after transitioning, you visit them as before, like Dr… used to. That will be better for people. People even will like to change for them. [IDI_female patient general clinic_ESE_23]
Family support therapy	01	I shared this with one family member, and, she asked me about what the doctor had told me, about the new drug… because she is supporting, she was like okay lets us see, if you see anything, you talk to me. [IDI_male patient_general clinic_ESE_11]
Expert peer involvement in drug switching	01	Those that have experienced switch… facilitate them to share about what they have been though… they really prepare their hearts to be expectant. (IDI_male patient_ peer leader_ESE_9]
Counselling about drug switching	05	…. they tell them when they are together, the counsellor comes and talks to them, “we are changing this because of this and this” … [IDI_female patient_ general clinic_SOD_8]
Capacity building for staff at all levels who switch drugs	01	People need… refresher training. Actually many, many people by the countryside, Yeah! Many of those Health Centres at lower levels; they are not yet there… [IDI_male patient_ Co-pay _SOD_16]
Monitoring DTG side effects	04	They need to carry investigations to make sure this person the kidney is either OK or liver; so as not to give a drug, which again worsens someone’s condition. [IDI_male patient_Co-pay_SOD_16]
Community sensitization through community meetings	12	I say there should be doctors moving around sensitizing about the issue… doctors sensitize people during community gathering about that situation… [IDI_female patient general clinic_SOD_22]
Put up information charts about drug switching	04	I do not know how they call it, these charts; you put them up at the waiting area… within the facility here. Some will get interested and start reading and it… [IDI_female patient _co-pay clinic_SOD_25]
Routine health talks about switching	06	Teaching those that came early at 8am, and then another health worker comes in to teach at 11am after the others have come. Most times if you do not come early, you do not even know what is going on. [IDI_female_patient_RSOD_24]
Routine viral load monitoring after switching	03	I mean the viral load testing should be done after a year. Eh, how do I know that it is suppressing…. Whenever I come I ask, “are you taking my viral load, are you taking my viral load?” “No! October….” [IDI_female patient general clinic_SOD_8]
Adequate messaging and explanation about switch benefits	02	I wanted to know why they had changed it to this new drug. Was it any better compared to the other 3 tablets? Was it reducing on the inconveniences of taking it? and the benefits of this new drug. Was it leading us to the discovery? I would have loved to hear that. [IDI_female patient_SOD_1]

## Involvement of sexual partners and care givers in patients rushed decision to switch

Most participants said they shared information given to them by health care providers about switching to a new treatment option to their care givers, who included elder children, elder siblings, spouse, uncle, or mother. Participants said they mainly shared Information given by health care providers during switch such as benefits of switching to DTG, the expected side effects and how to take it to DTG. Most care givers were willing to support patients in any way possible:

I shared this with my elder daughter, she asked me about what the doctor had told me, about the new drug and I told her exactly what the doctor had told me and then... because she is supporting, she was like okay lets us see, if you see anything, you talk to me. [IDI_male patient_ general clinic_ESE_11]

Some participants said they consulted their ART experienced family because they were not satisfied with the amount of information given by health providers during the clinic visit. They found such ART experienced family members understanding, supportive. They received confidence to take the new drug when family members shared positive experiences about their own switch:

...apart from a few I share with like there is one also who, he is my paternal uncle, he gets from here. When they gave me the drug I asked him, “Have you seen this change there? What is taking place? What is wrong?” because he picks from here. So whenever I get like a change, I first call him and find out... I get more confident... I take even a photo then I send him, I say, ‘they have changed me on this... [IDI_female_patient_SOD_8]

Some participants said their spouses were happy about the medication change. When a spouse received proper explanation about the switch, they offered adherence and social support during switch to a new drug:

My husband has no problem, I returned home and I told him that they had changed my drug and he said okay. He supports me on everything. Me and him we swallow on time. Our drugs, are kept together, and our boiled water is available...as long as I have explained to him properly, he supports me... [IDI_ female patient_RSOD_18]

One participant said she wanted to consider changing her decision about the new drug when she suffered side effects but received support and courage from her spouse to take it. Her spouse also encouraged her to report any side effects resulting from the switch to a new drug:

So I explained to my husband; he is the one that sent me, he said ‘go! Look at how swollen your face, arms and legs are. Go and explain to them.’ That is when I came. I had refused to continue with it but I said let me go ahead. He said, ‘no, you may collapse without knowing, you hear me? Go there.’ Then I started worrying about that new drug... what is wrong? [IDI_female patient general clinic_ESE _23]

Some participants said their male sexual partners supported switch because they liked the idea of changing to a fixed dose. One participant said her spouse accepted HIV testing because the new ART option would be easy for him to take:

He didn’t feel bad about it. He said, ‘that one also; at least if they test me’ because he has never gone for testing, ‘if they find me with the virus, this drug will be easy for me’ (IDI_female_ general clinic_SUT_4)

Some female participants said their husbands were uncertain as to why drugs were changed, yet their wives were stable on their previous regimen. The perception that switching to a new treatment option was due to poor adherence scared some spouses:

When I was telling him and he got scared, he thought I was adhering poorly so the other drug was not working for me and that is why they gave me another one. [IDI_female patient_ general clinic_SOD_17]

## Suggestions by patients for proper communication and effective orientation to new treatment option

Most participants said updated information flyers or leaflets should be distributed to all patients upon arrival at the health facility, to communicate and orient all patients to DTG. Information flyers or leaflets should include DTG benefits, related side effects, a convenient time to take it, and nutrition requirements. It should be in appropriate languages and include contact to report side effects. Patients are able to read for themselves and go back at home to read and reflect further on DTG:

I think they should also start making flyers; we should go back at home and read about the whole thing that this drug is this and this... even reminding people about the time, not to miss... you have to eat well... [IDI_female_patient_SOD_8]

Place illustrative charts and attractive information posters at different service points such as reception, pharmacy, clinical areas, to interest patients to read about benefits of the new drug and what to do in case they experienced side effects:

... put up these charts; like where we get the drugs from, where people line up, within the facility here. Some will get interested and start reading and it will be also received. [IDI_female patient _co-pay clinic_SOD_25]

Avail newsletters or bulletins periodically for patients to read about new treatment options, benefits and management of related side effects:

Maybe supply newsletters... for the people who can read, yeah even videos in different dialects especially English and Luganda...short videos. [IDI_male patient_co-pay clinic_ SOD_15]

Some participants suggested short DTG demonstration videos of less than 10 min to be played on a television screen for patients to watch as they wait to be served. Audio visual recorded Information about DTG benefit, management of side effects, and, statistics of what is happening elsewhere in the world regarding DTG should be included. Information should be recorded in English and translated in other common local language:

People watch DTG video clips... One of you or two or three, and the people who have started discussing these things am sure may be ten-minute video. [IDI_male patient_co-pay clinic_ SOD_15]

Home visitation to be done by health care providers to monitor patient’s health, offer psychosocial Support and avail incentives such as food and finances.

... They changed my drugs… they came home… You know here we got so many friends, you see. “They changed my drugs, they visited me, brought me bread and even gave me shs. 20,000” (equivalent of 5 US$)) … [IDI_female patient general clinic_ESE _23]

## Discussion

Understanding patient experiences of provider-initiated ART switching is important to developing intervention strategies to improve future ART switches. In this qualitative study, we found that most patients did not object to switching to DTG when encouraged and supported by their providers. We also found that most patients who had switched from EFV to DTG were glad they had done so. Transition of medications in public health programs represents specific periods of heightened information asymmetry. During this time, health care workers (HCWs) have much more information about the new drugs, and patients may feel disempowered if they still have unanswered questions at the time the switch is performed. Information pertaining to people starting ART may be different from those switching from their ART regimen to another [21]. Notably, information needs vary from patient to patient. There is need for sufficient, reliable and adequate information about risks and benefits of switching to DTG among people already on ART [9]. Some patients were concerned about questions related to dosing, adverse events, and reproductive needs (Additional file 1, Additional file 2).

Despite finding that participants had some unanswered questions about DTG switch, most of them did not object to switching to DTG when encouraged and supported by their providers. Most participants who had switched from EFV to DTG were glad they had done so because the switch resolved EFV-related side effects, including dizziness and hallucinations. Most participants liked the once-daily morning dose and the small pill size of DTG-based ART, although many said they struggled to adjust to a new dosing routine. Participants enjoyed switching to a drug that was more beneficial as per MoH recommendations. This study builds on previous research in which researchers showed that DTG is superior to existing treatment regimens and has a more tolerable side-effect profile [3]. Drug switching was driven by the potential direct health benefits of DTG to patients over current first line EFV-based regimens [9], including increasing pre-treatment resistance to NNRTIs, fewer side effects, rapid viral suppression and low pill burden [22]. Pill burden (number of tablets taken daily or large tablet size) is associated with suboptimal adherence [23]. Thus, patients may have better adherence with DTG because of its reduced pill burden.

Most reports have indicated improved tolerability with DTG versus previous EFV regimen with substantial reductions in the number of adverse events. However, some patients experienced some psychiatric effects after switch to DTG as expressed by a participant who said, “I felt like running mad’’. Other participants reported depression, dizziness, and headache which resulted in treatment discontinuation. Since it has been demonstrated that experiencing side effects may result in poor adherence [24, 25], there is need for support from health care providers to encourage patients to report any unusual event from the drug, manage toxicities as well as offer adherence support.

We found that women with childbearing potential were concerned about neural tube defects from DTG exposure [21]. Drug substitution may confer new side effects, which calls for ongoing information sharing and ART adherence support [6]. Data from Botswana suggested that periconception use of dolutegravir was associated with an increased risk of neural tube defects [26, 27]. This safety concern led to precautionary alerts from WHO and other regulatory agencies, and the restriction of use of DTG in pregnancy or by women of childbearing potential who were not using effective contraception [27], which delayed implementation of DTG roll-out [28]. Although initial studies suggested a possible link between DTG and neural tube defects, data from clinical trials comparing the efficacy and safety of DTG and EFV in Africa showed that the risk of neural tube defects was significantly lower than initially thought [11]. A randomized trial found that DTG was safe and effective in women and their infants when DTG-ART was initiated in the third trimester with follow-up until 72 weeks postpartum [27]. Disseminating correct information about the lack of association between DTG and neural tube defects is key for participants to accept switching. Programmes should continue strengthening pharmacovigilance monitoring of DTG related adverse events as well as birth outcomes.

Participants simultaneously felt unprepared, and worried about DTG-related side effects including insomnia, headache, and weight gain, which negatively affected their quality of life and adherence to the new regimen. Adherence difficulties occurred after switching from EFV nightly dosing to DTG morning dosing. All participants struggled with adherence including selecting dosing times compatible with their work schedules. Given that this was a health provider-initiated switch, there is need to adequately monitor changes in routine of taking drugs for participants. Health providers need to re-emphasize the importance of adherence to the switched drug to avoid poor adherence that may result in treatment failure. The side effect commonly explained by health providers was trouble sleeping. This is in agreement with other studies that reported adverse events that tended to occur soon after starting new medication and improved over time [13]. There is need for careful monitoring of DTG since side-effects not previously reported in early clinical trials, such as weight gain, have subsequently been reported from observational studies and randomised trials [29–31]. The programmatic roll-out of DTG-based first-line ART should prioritize effective diabetes prevention and treatment strategies for patients switched to DTG [32]. Our study suggests that giving short weekly return appointments to participants helps to monitor drug switching and manage possibly-related side effects. Participants should be educated about management of possible side effects following DTG switching and to seek help from a HCW when they experience side effects. Calling a toll-free number given to participants by HCWs was a mechanism through which they felt free to report drug related challenges including side effects. Participants were also encouraged by health providers to have a phone contact for a specific doctor to report side effects. Some switched participants benefited from support of significant others to manage side effects.

Previous findings revealed a tension between the rapid pace of transition and the weak health system preparedness of DTG switch that resulted in the development of tentative guidelines with no robust evidence to support critical decisions [9]. Delivery of information was done through counselling sessions, clinical sessions and health talks. However, some sessions may have been too brief for clients to address all the questions that could have emerged after the session was completed or even at home. Furthermore, some clients could have missed the talks. The majority of participants felt they had to accept the switch to DTG because it was government policy. Prior research suggests that decentralized ART-programmes need close support, supervision and mentoring to absorb new treatment guidelines and to adhere to them [11]. Our study suggests that adequate planning and preparation by health providers is needed in order to implement drug switching as per required new guidelines. Future switches merit a gradual approach that allows for all the necessary institutional and individual capacities to be strengthened to adequately support switched patients [9]. Switching to a new drug requires comprehensive planning and adequate preparation prior to implementation.

During transitions, programs should consider creating avenues for a “pull system” or “on demand” information about new medication, by increasing access to reliable information within (audio-visual recordings playing throughout the day to complement ongoing education and counselling activities), and outside the health facility e.g. toll-free phones or via community engagement models. Participants may be informed about new drug switch guidelines and mechanisms to report drug switch related issues through group or individual counselling sessions at the health facility. Recent communication and integrative models of shared decision-making offer promising approaches for helping to address key decision-making constructs [33]. Health systems should develop mechanisms to record experiences from previous transitions, in order to inform future transitions and reduce repeated mistakes [9]. Communicating to participants about reasons for drug switching as well as explaining possible DTG side effects in a provider-initiated switch is important as it enhances their informed decision-making.

## Strengths

Our study is one of the first qualitative evaluations of patient experiences of health provider-initiated switch to DTG-based ART while on a stable NNRTI regimen in sub-Saharan Africa. Our study provides insights on how participants were prepared to adhere to MoH switch guidelines. This study was done at a single large urban center of excellence for HIV care where switch support was available from well-trained and experienced providers.

## Limitations

Our study has some limitations. The key limitation of the study is that the IDI clinic is a center of excellence with several doctors and clinicians with several years of experience in HIV care unlike most HIV care facilities throughout the country, limiting the generalizability of our findings. The results of this study therefore show that gaps exist during rollout of new drugs even in a well-established facility. Thus, switching to DTG-based regimens would likely be suboptimal in less resourced health facilities, including in rural areas. Nevertheless, these emerging data from a large tertiary care clinic are an important contribution to the field of drug switching. Ascertaining patterns of drug switch in rural settings will be critical to inform HIV programs when scaling up new HIV treatments. Second, our results are an initial account of DTG-switch experiences that limit perspectives over time. Finally, the drug transition was similarly challenging for participants who pay for their ART services, suggesting that effective communication is needed prior to drug switching irrespective of socio-economic status.

## Conclusions

Amongst adults with HIV and stable on an NNRTI regimen but switched to DTG regimen in a program-wide policy change, participants generally preferred the DTG-based regimen because of the small pill size, once-daily dosing, as well as the absence of EFV-like side effects. Participants, however, found the time between being counselled and switched inadequate, and would have preferred more time to understand the DTG regimen and the implications of drug switching. Ensuring proper communication to patients switching to a new treatment option requires collaborative effort to develop effective communication interventions. There is need to conduct research among health care providers and other stake holders to understand how to communicate switch to a new drug and address the support they need to switch patients to a new treatment option. Community-engaged research is needed to devise more acceptable ways to prepare participants for switching ART especially when done at a program wide scale.

## Supplementary Information

**Additional file 1.** Topic guide for indepth interview (IDI) with patients ( Version 1.4) February 26, 2019. Note: Topic guide in the manuscript is Under data collection.

**Additional file 2.** Table of a DTG Code manager extracted from ATLAS.ti Version 8. Note: Code manager in the manuscript is under data management and analysis.

## Data Availability

Data analyzed for this manuscript are available from the corresponding author upon request.
