# Supplementary material for: Patient experiences of switching from Efavirenz- to Dolutegravir-based antiretroviral therapy: a qualitative study in Uganda
Source: BMC Infect Dis. 2021 Nov 13;21:1154. doi: 10.1186/s12879-021-06851-9 (PMC8590364; doi:10.1186/s12879-021-06851-9)
Supplement: Supplementary file 1 — Additional file 1. Topic guide for indepth interview (IDI) with patients ( Version 1.4) February 26, 2019. Note: Topic guide in the manuscript is Under data collection. [file 12879_2021_6851_MOESM1_ESM.docx]

# **Appendix E: Topic guide for in depth Interview (IDI) with patients (Version 1.4 February 26, 2019).**

**Study Title**

**PATIENT’S EXPERIENCES OF SWITCHING FROM EFAVIRENZ (EFV) TO DOLUTEGRAVIR (DTG) - BASED ANTIRETROVIRAL THERAPY IN UGNDA.**

Date;

Interviewer's name

# ID No

Community/town

Gender: Male
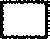
 Female
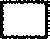


**Introduction**

Good morning /good afternoon my name is...... And we are conducting a study exploring how and why people decide to change from EFV to DTG. I will be asking you about your views and experience regarding such changes when it comes to HIV drugs. This interview will last for about one and a half hours. There is no right or wrong answers am just interested in your experiences, views and opinions. So please feel comfortable to say what you think and to give your honest views and opinions I also request you to kindly allow me to tape record the interview as this will help me to accurately capture all information. The recordings from this interview will be kept confidential and will only be available to the research team. If at any time during the interview you feel uncomfortable you can ask for a break, refuse to answer any question, and are always feel free to leave. Do you have any questions before we start?

# **Warm up (socio-demographic information)**

1. Could you please start by telling me a bit about yourself?

- PROBE: for how long they have been attending the clinic?
- PROBE: How far they have you travelled?
- PROBE: How are you, 18- 24yrs
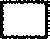
 < 24years
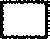

- PROBE: Are you married or single?

Married/living with partner
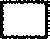
 Separated
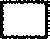
 Widow
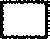
 Single
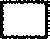


- PROBE: What is your level of education?

Never gone to school
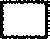
 Primary
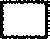


Secondary
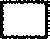
 Post-secondary
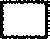


- PROBE: What do you do for a living?...........................................................
- PROBE: Which ART regimen do you currently use or are currently on? Efavirenz dolutegravir
- PROBE: Were you using a different ART regimen before?

Yes
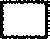
 No
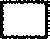


## **Drug substitution**

1. Tell me about your experience of starting ART?


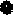
PROBE: When did you start, and why that time?


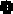
 PROBE: What were your reasons for starting ART?


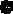
 PROBE: what are your thoughts on being on ART?


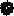
 Do you have any concerns about being on ART?


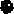
 PROBE: What motivated you to start ART?


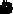
 PROBE: Risks and benefits of ART

1. Tell me how many times have you changed your HIV medication?

• PROBE: The first time which medication was changed and why?


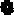
 PROBE: What it was replaced with


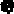
 PROBE: What was the reasons for the change?

Decision- Making Process

1. Tell me about your experiences the last time you changed your medication from the previous ones you were taking.
   - PROBE: who initiated the change in your medication and why was it initiated?
   - PROBE: how did you feel about the change?


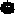
 PROBE: How did your partner and other family members feel about the change of your medication?

1. What information did you receive about the new drug and how helpful was it in helping you make a decision.
   - Probe about knowledge of the purpose of drug substitution e PROBE: When do you think it is ideal for a positive person to substitute drugs and? Why?
   - PROBE: What do you perceive as the benefits of changing drugs?
   - PROBE: What do you think are some of the risks?
2. Can you share with me what do you think can be done differently to enable patients change to drugs efficiently?
3. Tell me what you know about pregnancy in HIV?


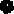
 PROBE: How does MTCT occurs (e.g. pregnancy, delivery and breastfeeding, etc.) and how it can be prevented.


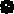
 PROBE: What do you know about values of ART in pregnancy and breastfeeding?


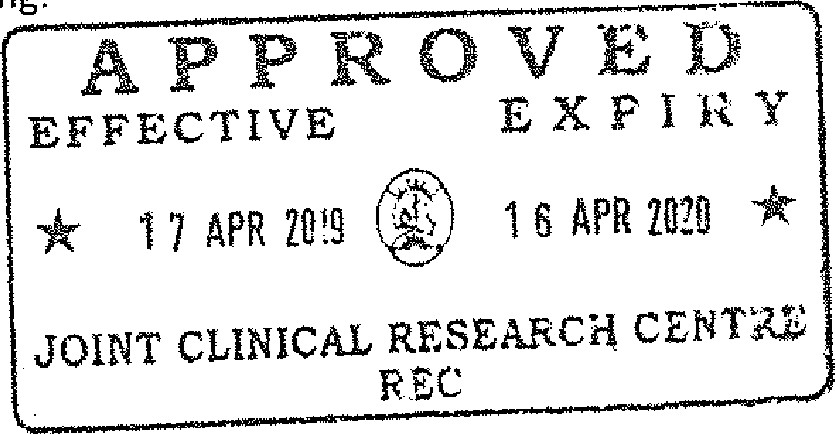


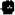
 PROBE: Whose health do they put first when it comes to decision about ART and why — baby or mother?

• PROBE: What information did the health workers tell you about DTG use and pregnancy? Probe about side effects, use of contraceptives.

1. There are suggestions that to replace EFV with DTG because it clears the virus rapidly from the blood, however it may be associated with birth defects (Neural Tube Defect) when used in the first few weeks of pregnancy (1st trimester). Would you use D TG if you were planning to fall pregnant? Why?


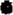
 PROBE: What does your spouse think about changing from Efavirenz to DTG in pregnancy?

- - - PROBE: What do your Family members think about changing from Efavirenz to DTG in pregnancy?
    - PROBE: What does your friends or community member's think about changing from Efavirenz to DTG in pregnancy?


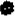
 PROBE: Would you accept or consider use of DTG in case you are on long term contraceptives and why?

- - - PROBE: How are decisions made about contraceptives in your fife, who decides when and what methods to use for contraceptives. Probe for the Role of patients, spouse, friends and health .workers)

PROBE: Would you accept or consider use of DTG in fate pregnancy and why?


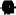


Is there anything else you want to tell us about changing from EFV to DTG? **Thank you for your time and your valuable information.**
