# Supplementary material for: Patient experiences of switching from Efavirenz- to Dolutegravir-based antiretroviral therapy: a qualitative study in Uganda
Source: BMC Infect Dis. 2021 Nov 13;21:1154. doi: 10.1186/s12879-021-06851-9 (PMC8590364; doi:10.1186/s12879-021-06851-9)
Supplement: Supplementary file 2 — Additional file 2. Table of a DTG Code manager extracted from ATLAS.ti Version 8. Note: Code manager in the manuscript is under data management and analysis. [file 12879_2021_6851_MOESM2_ESM.docx]

**Code Manager _ DTG_ ATlasti version 8.**

| **CODE GROUP** | **Description** | **CODES** |
| --- | --- | --- |
| Previous experience on EFV or other regimen. | Negative experiences of EFV or other regimen as described by participants. | - Drug substitution reduce d EFV related side effects - Reason for switching from Combivir was because it was banned due to side effects. |
| purpose or benefits of Switch | What participants described as the purpose and or benefits of switching drugs | - DTG switch was mandatory - Government policy was the reason for switch - New drug innovation is the reason for switch - Not given a chance to ask about reason for switching - Don’t know reason for change - Does not remember reason for change of drugs - Cabinet decision about change of drugs - Changed to DTG because doctor said it was good medicine - called back to switch off DTG as per clinic requirement - changing drugs contributes to research - Drug side effects resolve with taking milk - drug substitution beneficial if adherent - drug substitution improves immunity - drug substitution prevents complications - Drug substitution reduces EFV side effects - Healthy life is a benefit of changing drugs - Reason for switch was to have a fixed dose formulation - Reason for switch was to relieve side effects - Reason to switch was to get smaller pill - switched to second line due to toxicities |
| Risks of switching drugs | What participants described as risks of drug substitution benefits of switching drugs | - Risk of a strong drug - Risk of switch is worsening condition - Rushed switch - change of drugs might lead to death - complications may result from substitution - concerned about community stigma after suffering side effects related to switch - Does not support switch to stable patient |
| knowledge gaps about DTG | Perception about DTG as stated by participants | - Don’t know about current DTG drug switched |
| Initiator of DTG switch | Person responsible for initiating switch and why was it initiated | - Doctor decided to switch to DTG - counsellor initiated switch - self-initiated switch |
| Inclusion and exclusion criteria for DTG switch | What participants said about who was eligible for switch | - file was flagged for switch - eligibility criteria for DTG was for women who did not need to have more children - Eligibility for DTG switch is viral load suppression - Eligibility for switch is perfect adherence - Eligibility criteria for DTG is use of FP - Explain reason of switch - child bearing age was an exclusion criteria - post-menopausal |
| Patient/ provider Interaction or relationship during DTG switch | Description of Patient/ provider Interaction or relationship during DTG switch | - perceives IDI as a center of excellence that promoted switch through a multidisciplinary team - pharmacists explained how to take drugs - Received adherence counselling - Received counselling about the dietary restriction of DTG - Receptionist explained switch |
| Perception about information shared about switch, risk or benefit communication about DTG by health providers | Description about information received during switch and how helpful was it in helping you make a decision. | - information given made her decide to accept DTG - Not informed about the risks( side effects) of DTG by providers - Did not receive information about DTG switch and family planning - counselor quickly explained about switch to DTG - Received information about DTG benefits - Received information about FP - received information about side effects - Received information about timing of DTG. morning dose explained - Received storage information |
| Right time to switch to DTG | What participants mentioned was the ideal for a positive person to substitute drugs | - good to change incase difficult to swallow - good to change in case of treatment failure - Good to change if new drug has no related side effects - Does not know right time to change drugs - Right time to change is when someone suffers side effects - Right time to switch is if doctor perceives it beneficial - Right time to switch is when the viral load is high |
| Frequency of drug switching | Number of times and reasons for switching HIV medication as described by participants | - changed drugs thrice - Changed drugs twice - Changed once |
| Acceptability of DTG switch | Participant’s consideration of accepting DTG Switch. | - Accepted DTG switch due to related benefits - Accepted of switch to DTG during late pregnancy - Accepted Family planning to be able switch to DTG |
| Likes about DTG compared to EFV | What do you perceived as the advantages of switching to from EFV to DTG based regimen? | - DTG better than EFV - Changed to a superior new drug - Felt DTG was stronger than previous drug - headache stopped after switching off DTG - Nails normalized after DTG switch - CD4 rise is the benefit of switch - liked DTG reduced pills - Liked smaller pill - likes fixed dose formulation - Likes morning dose - likes once a day dose - likes small pill box for DTG - likes tiny tablet - Free new drug availed - Does not have any drug contraindication - DTG enhances viral load suppression - DTG has less side effects - DTG is easy to swallow - DTG is okay - DTG leads to weight gain - DTG more friendly to swallow - DTG resolved itching - DTG stopped secretions - Got few cases of people complaining about DTG |
| Dislikes of DTG | What do you perceived as the disadvantages of switching to from EFV to DTG based regimen? | - Suffered DTG side effects - suffered body itching after DTG switch was switched back to previous EFV regimen - DTG weakens the body - dislikes big pill - dislikes colour change - Dislikes conditions of use of FP to use DTG - Does not like brown colour - does not like change to a bigger DTG fixed pill - Suffered dizziness on DTG - suffered EFV hallucinations - suffered headache on DTG - suffered Nausea upon DTG start - DTG caused joint pains - DTG caused Neural tube defects( deformed children) - DTG causes Diabetes - DTG causes dizziness - DTG causes lack of sleep - DTG causes loss f appetite - DTG causes urine stones - loss of sight due to drug substitution |
| Response to side effects resulting from switch | What participant said they responded when suffered side effects | - given phone contact by health care workers to report DTG side effects - Switched off DTG. - Received short return appointment for monitoring - Returned to IDI to report side effects - consult doctor about DTG use in late pregnancy - coped with dizziness by eating a lot - coped with side effects by drinking water - coped with weight loss by changing locations - Support from partner was sought - Received short return appointment for monitoring - DTG side effects resolved after switching back from DTG to previous regimen |
| Adherence and DTG switch | Description of how switch may influenced drug adherence | - Fixed dose promotes good adherence - Change of time for new drug might affect adherence - poor adherence is the risk of switching drug |
| Informed choice about DTG switch | What the participants mentioned about informed choice about switch | - signed indemnity form to receive DTG |
| Pregnancy and DTG switch | Perception about considering DTG switch if pregnant or planning to fall pregnant | - Accepts DTG in late pregnancy - Accepts DTG after birth - Agreed to use FP to use DTG - Uncertain about effect of DTG in pregnancy - Concerned about Botswana study that revealed results about risk of DTG issue with women of reproductive age. - Desires to conceive while on DTG - Disapproves DTG in late pregnancy |
| Family support during switch | How participants partners and other family members perceived switch from Efavirenz to DTG | - Family member supportive of switch - Got uncle support for switch - mother supported switch - Spouse supported - spouse complains of forgetfulness after switching to DTG - Spouse encouraged her to report - spouse initially refused DTG switch - side effects to health workers |
| peer influence | How peers perceived participant’s switch from Efavirenz to DTG | - client say wrong information about DTG - Discussed with peers about DTG side effects - Some peers had similar DTG side effects and had been switched back to previous EFV regimen - Decided not to hand over previous drugs prior to switch after peer advice. |
| Concerns about switching to new drugs | Concerns about switching to new drugs as mentioned by participants | - Not given enough time to prepare for switch - switched by surprise - Rushed switch - patients worry once they read about side effects on pamphlets - Hesitancy to switch - People may reject to switch to another drug once side effects are mentioned. - Perceives doctors lie to patients to convince them to switch drugs. - negative perception abut switch in the community - Found multiple drug switching weird - initially was not told about DTG side effects - Fear for switch was if body took time to adjust - Fear of affecting child is the reason for disapproval of DTG in pregnancy - felt weird to change yet previous regimen treated good - got scared after DTG switch - Uncertain about effects of witch switch - Scared to get off DTG yet desires to conceive |
| desired information during switch initiation | What the participants described as the desired information during switch initiation | - inform patient about drug name - Explain to patient’s about minor symptoms of DTG - explain to patients that symptoms disappear with time - Wanted to know if new drugs were better compared to others - Explain about how new drug works - Explain to patient about reason for changing drugs. - Specific demonstration about how new drug works. - wants choice information to choose between DTG - Explain risks and benefits of drugs - Explanation about dietary restriction is key - Explanation about switch process is key |
| Suggestions to improve switch | What participants said can be done differently to enable patients switch drugs efficiently. | - Put up information charts - put up news letters - put up newsletters - Radio and TV sensitization - show videos - Read information about DTG side effects on pamphlet - Organize seminars about switching drugs - Use expert peers who have switched - Reduce size of DTG - refresher training key for staff switching - Research is key prior to switch - Viral load monitoring key 3months after switch - monitoring adverse events key - monitoring urine key - Having a phone contact to report side effects is key - counselling for positive living is key - counselling for adherence - change of drug should depend on patient health condition - clear information be given to patient to base their decision to switch - community sensitization - community support for patient is key - avail transport refund and lunch to patients eligible for switch - Blood check is key in drug substitution - couple support for adherence during switching drugs is key - Desires cure for HIV - desires HIV vaccine - Desires long acting ART - Following doctor's advice is key - Give out information flyers - good attitude of health workers - Good diet is key in drug substitution - Home visitation by doctors is key during switch. - Strengthen Health talk - Lab equipment key in substitution - Pre- switch counseling is key - Prefers injection compared to tablets - prefers smaller tablet - Prefers white tablet - preparing patients for switch is key |
